# Effects of transversus abdominis and erector spinae plane blocks on postoperative pain control and postpartum depression after cesarean section: a randomized prospective study

**DOI:** 10.55730/1300-0144.6155

**Published:** 2026-01-19

**Authors:** Hilal BİRADLI, Feyza ÇALIŞIR, Alev ÖZER

**Affiliations:** 1 Department of Anesthesiology and Reanimation, Faculty of Medicine, Kahramanmaraş Sütçü İmam University, Kahramanmaraş, Turkiye; 2 Department of Obstetrics and Gynecology, Faculty of Medicine, Kahramanmaraş Sütçü İmam University, Kahramanmaraş, Turkiye

**Keywords:** Cesarean section, erector spinae plane block, obstetrical anesthesia, postoperative pain, postpartum depression, transversus abdominis plane block

## Abstract

**Background/aim:**

Effective postoperative pain management after cesarean section is essential for maternal recovery. Poor pain control can lead to complications, including postpartum depression. This study aimed to compare the postoperative analgesic efficacy of erector spinae plane (ESP) and transversus abdominis plane (TAP) blocks following cesarean section, with a particular focus on their potential impact on postpartum depression.

**Materials and methods:**

Sixty patients were randomly assigned to receive either an ESP block (Group E, n = 30) or a TAP block (Group T, n = 30) after cesarean section. Pain severity was assessed using a visual analog scale (VAS), and the need for rescue analgesics and patient satisfaction were recorded. Postpartum depression was evaluated using the Edinburgh Postpartum Depression Scale (EPDS) at 4–6 weeks postpartum.

**Results:**

There was no significant difference between the groups in analgesic duration (Group E: 15 h, Group T: 14 h, p = 0.314). Group E showed a significantly lower need for rescue analgesics (0 vs. 1, p = 0.049). The VAS score at the first hour was lower in Group E (2 vs. 3, p = 0.032), but no differences were observed at subsequent time points. Postpartum depression rates were not statistically significant.

**Conclusion:**

With the ESP block, no significant difference was observed compared to the TAP block in terms of total analgesic duration, opioid consumption, postpartum depression, or patient satisfaction, except for the postoperative 1-h VAS pain score. This suggests that the ESP block does not provide clear superiority over the TAP block.

## 1. Introduction

Successful pain management after a cesarean section positively impacts the patient’s recovery process and clinical outcomes. Inadequate pain control can restrict movement and negatively affect the healing process, leading to complications such as thromboembolism, chronic pain, and postpartum depression [1,2]. Effective pain management after a cesarean section is also crucial for proper care of the newborn, prompt establishment of mother–baby bonding, and successful breastfeeding [3].

Opioid analgesia administered intravenously, while effective, carries risks such as transfer to breast milk and respiratory depression. Therefore, multimodal analgesia techniques that reduce postoperative opioid use are emphasized. Regional anesthesia techniques such as transversus abdominis plane (TAP) block, erector spinae plane (ESP) block, lumbar paravertebral block, quadratus lumborum block, and ilioinguinal-iliohypogastric nerve block are commonly used for analgesia after cesarean sections. Nonopioid systemic analgesics such as paracetamol/acetaminophen and nonsteroidal antiinflammatory drugs are also used for postoperative analgesia [4].

The TAP block was first described by Rafi in 2001 and was initially reported to block the 7th through 11th intercostal, subcostal, ilioinguinal, and iliohypogastric nerves [5]. However, both clinical and cadaveric studies have demonstrated that lateral TAP blocks primarily provide somatic analgesia in the abdominal wall, covering the T10–L1 dermatomes, without effectively blocking visceral pain [6]. The ESP block, first introduced by Forero et al. in 2016, involves the injection of local anesthetic between the erector spinae muscle fascia and the transverse process of the vertebra [7]. This technique has been shown to effectively control both somatic and visceral pain, making it a potentially superior alternative for multimodal postoperative analgesia [8].

Postpartum depression (PPD) is a significant psychological condition affecting a considerable proportion of women after childbirth. Studies have shown that inadequately managed postoperative pain may contribute to the development of PPD. Given that regional anesthesia techniques can significantly reduce pain levels, optimizing postoperative analgesia may play a role in reducing the risk of PPD [9,10]. However, data comparing the effects of ESP and TAP blocks on PPD are limited.

In this study, we aimed to optimize postoperative analgesia following cesarean section by comparing the effectiveness of ESP and TAP blocks under spinal anesthesia. We hypothesized that ESP blocks would provide superior pain relief compared to TAP blocks. Additionally, the potential relationship between these regional anesthesia techniques and postpartum depression was assessed.

## 2. Materials and methods

### 2.1. Ethical approval and study registration

This randomized prospective study was conducted in the Kahramanmaraş Sütçü İmam University Faculty of Medicine following approval from the Kahramanmaraş Sütçü İmam University Faculty of Medicine’s Clinical Research Ethics Committee on September 20, 2023 (Approval No: 2023/10-01). The study was registered at ClinicalTrials.gov (NCT06221280) and adhered to the 2013 Declaration of Helsinki and Consolidated Standards of Reporting Trials (CONSORT) 2010 guidelines. Informed consent was obtained from all participants before enrollment.

### 2.2. Study design and participants

This was a prospective, single-center, randomized controlled trial involving obstetric patients who underwent elective cesarean section under spinal anesthesia between September 2023 and May 2024. The inclusion criteria were age of ≥18 years, gestational age of ≥36 weeks, and American Society of Anesthesiologists (ASA) II–III classification. The exclusion criteria included patients in labor at term, those with obstetric emergencies, twin pregnancies, diagnosed psychiatric disorders or use of psychiatric medications, local anesthetic allergies, peripheral neuropathy or neuromuscular disease, anticoagulant use, contraindications for spinal anesthesia (e.g., coagulopathy, severe hypovolemia, increased intracranial pressure, infection at the injection site), body mass index (BMI) value of ≥35 kg/m^2^, or issues with the patient-controlled analgesia (PCA) device within the first 24 h postoperatively.

### 2.3. Randomization and blinding

Patients were randomized using a sealed-envelope technique with a 1:1 allocation ratio. Thirty patients were assigned to receive ESP blocks (Group E) and another 30 received TAP blocks (Group T). The randomization sequence was generated by an independent investigator and the envelopes were opened in the operating room. To ensure consistency, all blocks were performed by a single anesthesiologist who was not involved in the postoperative pain assessments. The outcome assessors responsible for evaluating pain based on visual analog scale (VAS) scores and postpartum depression based on Edinburgh Postpartum Depression Scale (EPDS) scores were blinded to the treatment allocation to minimize assessment bias.

### 2.4. Patient enrollment and data collection

Patients were evaluated preoperatively for baseline characteristics including age, height, weight, BMI, ASA score, comorbidities, gestational week, parity, and APGAR scores at 1 and 5 min. In addition, preoperative EPDS scores were assessed to establish baseline depressive symptom levels before surgery. The EPDS, a validated screening tool consisting of 10 questions with a total score of 30, was used to assess PPD before surgery and between the 4th and 6th weeks postpartum. A score of ≥13 on the EPDS was used as the threshold to identify postpartum major depression [11]. The total time required to complete the scale was less than 5 min.

### 2.5. Block procedure

Patients adhered to standard fasting guidelines before surgery and were monitored intraoperatively with an electrocardiogram, noninvasive blood pressure measurement, and pulse oximetry. Under aseptic conditions in the sitting position, an intervertebral space (L3–L4 or L4–L5) was accessed with a 25-gauge pencil-point atraumatic spinal needle and 10 mg of hyperbaric bupivacaine was administered intrathecally. Sensory block level (T6) was confirmed using the pinprick test and motor block was evaluated using the Bromage scale. After surgery, bilateral ESP or TAP blocks were performed under ultrasound guidance.

### 2.6. Erector spinae plane block technique

**Figure 1:**
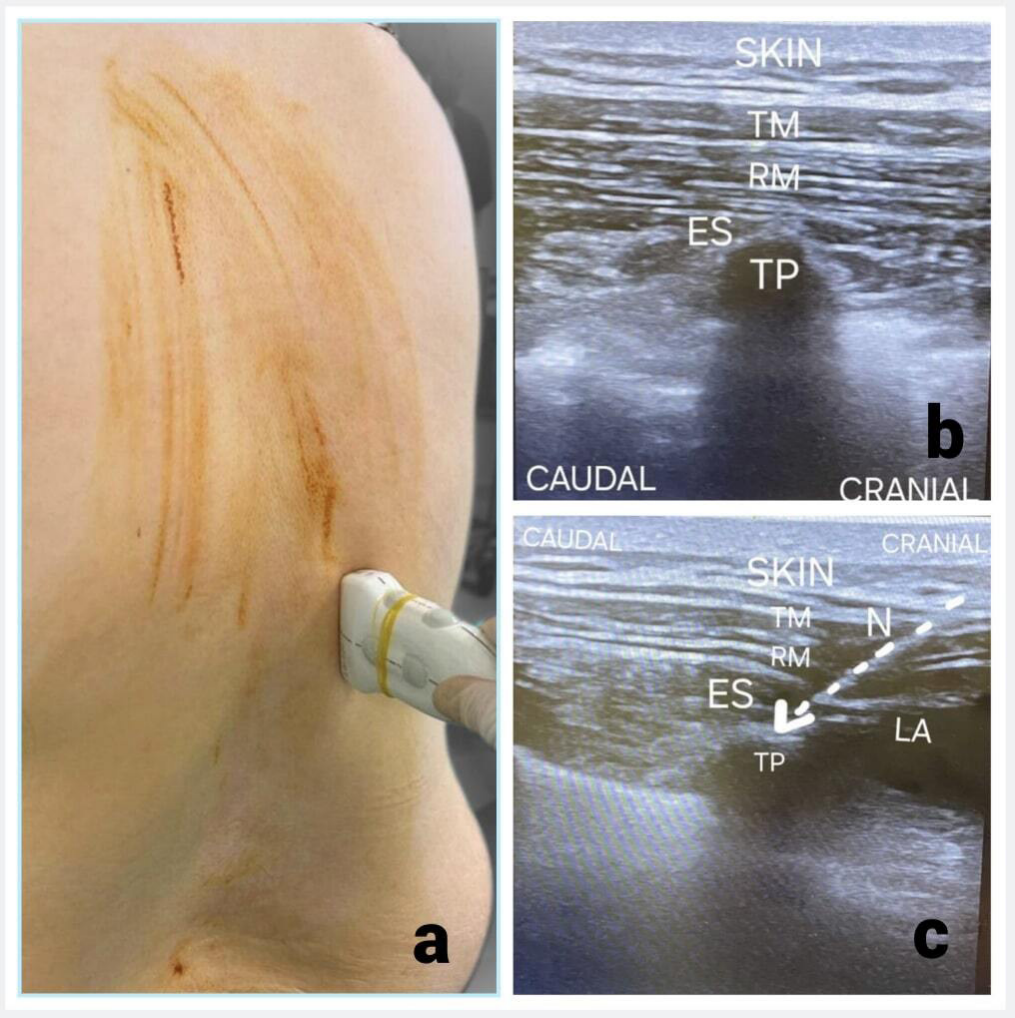
Erector spinae plane block technique from T11 vertebrae level (a). The needle is advanced from a cephalad to caudal direction; it is passed through the trapezius muscle (TM), rhomboid muscle (RM), and erector spinae muscle (ESM) until reaching the transverse process (TP) (b). At this location, an injection results in a linear spread of local anesthetic, causing the ESM to be displaced inferiorly (c)

For the ESP block, patients were positioned in the lateral decubitus or sitting position (Figure 1a). A 100-mm, 22-G block needle (Stimuplex^®^ Ultra 360^®^, Braun, Kronberg, Germany) was advanced in-plane from T11, 3 cm lateral to the spinous process, into the fascial plane between the erector spinae muscle and the transverse process (Figure 1b). After confirming negative aspiration, 20 mL of 0.25% bupivacaine was injected bilaterally with ultrasound monitoring (Logiq V1, 8–13 MHz, GE Healthcare, Shanghai, P.R. China) of the spread (Figure 1c).

### 2.7. Transversus abdominis plane block technique

**Figure 2:**
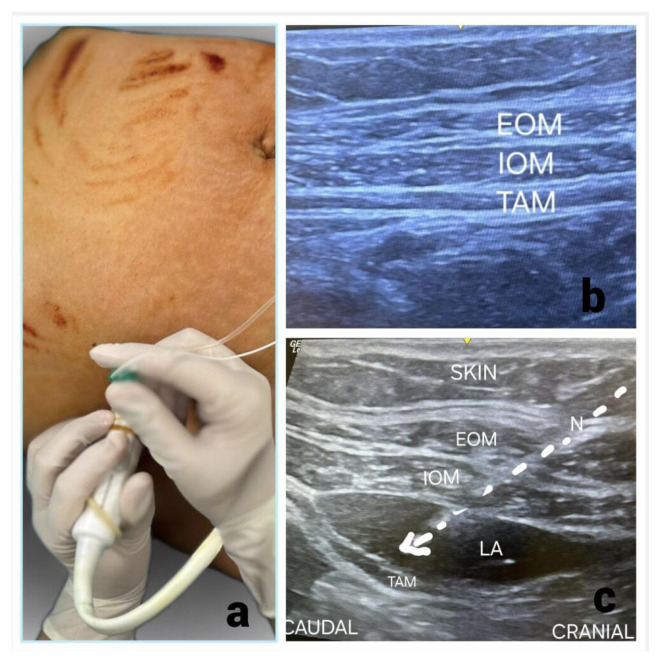
Lateral transversus abdominis plane block technique (a). The needle is advanced from a cephalad to caudal direction; it is passed through the external oblique muscle (EOM) and internal oblique muscle (IOM) until reaching the fascial plane below the transversus abdominis muscle (TM) (b). At this location, an injection results in a linear spread of local anesthetic, causing the TM to be displaced inferiorly (c).

For the TAP block, patients were placed in the supine position and a linear ultrasound probe (12–15 MHz) was positioned transversely on the anterolateral abdominal wall at the midaxillary line between the iliac crest and lower costal margin (Figure 2a). A 100-mm, 22-G needle was inserted in-plane through the subcutaneous tissues until the fascial plane between the internal oblique and transversus abdominis muscles was reached (Figure 2b). After negative aspiration, 20 mL of 0.25% bupivacaine was injected bilaterally, ensuring adequate fascial spread under ultrasound (Figure 2c).

### 2.8. Postoperative management

Patients received 1 g of intravenous paracetamol in the recovery room and were transferred to the ward with an intravenous PCA device (4 mg/mL tramadol, push dose of 20 mg, 20-min lockout, hourly limit of 50 mg, no infusion). Postoperative pain was assessed using a VAS (0–10 cm ), with patients indicating their pain levels on a continuous line between “no pain” (0) and “the worst pain of my life” (10). In this study, VAS scores of <3 were considered to indicate mild and tolerable pain, 4–6 indicated pain disturbing sleep but tolerable, and 7–10 represented severe, unbearable pain. Pain was assessed at rest, with the patient in a supine position and not engaging in any active movement. Pain during movement was assessed using a comfort scale (0–4 points), which was reversed in our study: 4 points indicated constant pain, 3 indicated a lack of pain at rest but severe pain on deep breathing or coughing, 2 indicated mild pain on movement, 1 indicated a lack of pain on deep breathing, and 0 indicated a lack of pain even while coughing. Assessments were performed at 0, 1, 6, 12, 18, and 24 h postoperatively. The 0-h evaluation was conducted in the recovery room immediately after surgery, while the patient was awake, hemodynamically stable, and in a supine position. Rescue analgesia (75 mg of diclofenac sodium intramuscularly) was administered if the VAS score was ≥4. The duration of block analgesia (time from block administration to first analgesic request) and total 24-h tramadol consumption were recorded. At 24 h, patient satisfaction was assessed using a four-point Likert-type scale (very good, good, moderate, poor), and complications within the first 24 h (itching, nausea, vomiting, headache, urinary retention) were noted.

### 2.9. Outcome measures

The primary outcome was total postoperative tramadol consumption (mg) within the first 24 h, recorded via PCA. Secondary outcomes included postoperative pain intensity measured with the VAS at 0, 1, 6, 12, 18, and 24 h; number of rescue analgesic doses administered; patient satisfaction levels; and postpartum depression incidence at 4–6 weeks, assessed using the EPDS (cutoff score of ≥13).

### 2.10. Sample size calculation

The sample size was calculated using G*Power 3 analysis software (Heinrich-Heine University, Düsseldorf, Germany). A preliminary pilot study was conducted with five patients per group, with postoperative tramadol consumption within the first 24 h recorded as follows: TAP group: 312 ± 138.56 mg, ESP group: 288 ± 72.11 mg. Based on these values, Cohen’s d was calculated as 0.95, indicating a large effect size. To test the superiority of ESP over TAP in reducing postoperative analgesic consumption, a power analysis was performed using statistical power of 95% (1 – β = 0.95) and a significance level (α) of 0.05. The minimum required sample size was determined as 25 patients per group. To account for potential data loss, 30 patients per group (total = 60) were included in the final analysis to maintain sufficient power for detecting clinically significant differences.

### 2.11. Statistical analysis

Quantitative variables were tested for normality using the Shapiro–Wilk test. Data were analyzed using IBM SPSS Statistics 22 (IBM Corp., Armonk, NY, USA). Continuous variables with normal distribution were compared using the independent-samples t-test, while nonnormally distributed variables were analyzed using the Mann–Whitney U test. Repeated measures were assessed using the Friedman test, with Bonferroni correction applied for post hoc comparisons. Categorical variables were analyzed using the chi-square test or Fisher exact test when applicable. Values of p < 0.05 were considered statistically significant. Data were expressed as mean ± standard deviation, median (Q1–Q3), number, and percentage.

## 3. Results

**Figure 3:**
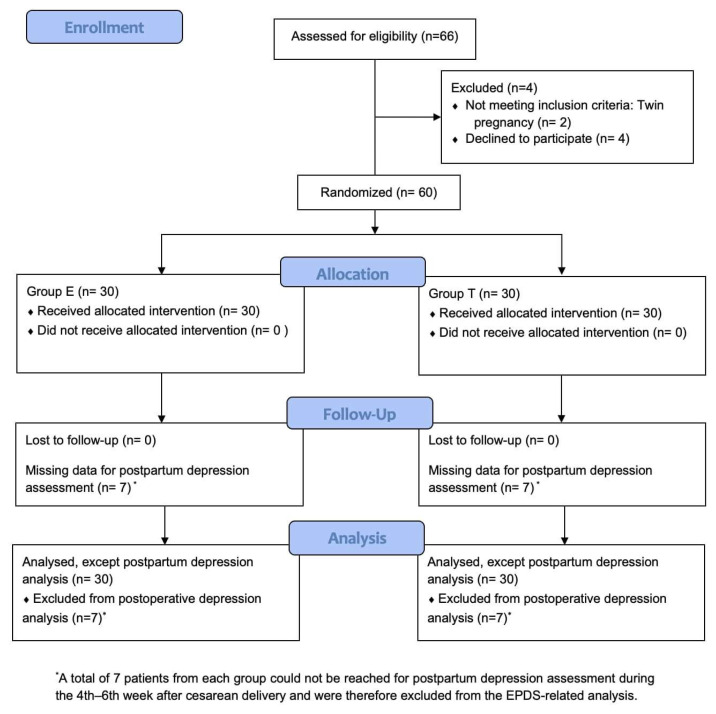
CONSORT flow diagram showing patient enrollment. Group E: Erector spinae plane block group; Group T: transversus abdominis plane block group.

A total of 66 patients were assessed for eligibility, with 2 excluded due to twin pregnancy and 4 declining participation. Thus, 60 patients were included in the study. For postpartum depression scoring performed with the EPDS scale, 7 patients per group could not be reached and statistical analysis for this outcome was conducted with 23 patients in each group (Figure 3).

Sociodemographic characteristics, including age, weight, height, BMI, ASA score, and education level, were comparable between the groups. No significant differences were found in comorbidities, pregnancy duration, number of pregnancies, APGAR scores, or operation duration (p > 0.05; Table 1).

Block analgesia duration was similar between the groups [Group E: 15 h (14–15), Group T: 14 h (14–15), p > 0.05]. Although total tramadol consumption over the course of 24 h was lower in Group E, the difference was not statistically significant (205 mg vs. 241 mg, p > 0.05). However, the number of rescue analgesic doses was significantly lower in Group E [Group E: 0 (0–1), Group T: 1 (0–1), p < 0.05].

Pain scores measured using the VAS showed significant changes over time in both groups (p < 0.001, Friedman test). Pairwise comparisons using the Bonferroni post hoc test indicated significant differences between baseline and multiple postoperative time points in both Group E and Group T (p < 0.05 for specific time comparisons). VAS pain scores were significantly lower in Group E at the first hour postoperatively [Group E: 2 (2–2), Group T: 3 (2–4), p < 0.05]. No significant differences were observed at subsequent time points (Table 2).

Patient satisfaction levels were comparable between the groups; however, the “very good” satisfaction rate was higher in Group E (73% vs. 57%, p > 0.05). No postoperative complications, including nausea, vomiting, itching, headache, or urinary retention, were reported in either group.

Preoperative depression rates were similar between the groups. Likewise, postpartum depression rates did not differ significantly (13% in Group E vs. 30% in Group T, p > 0.05). Postoperative EPDS scores at 4–6 weeks also showed no significant difference (9.4 vs. 9.8, p > 0.05) (Table 3).

## 4. Discussion

This randomized prospective study compared the analgesic efficacy of ESP and TAP blocks following cesarean delivery under spinal anesthesia. While ESP blocks demonstrated a statistically significant reduction in early postoperative pain (1-h VAS score) and rescue analgesic requirements compared to TAP blocks, no significant differences were observed in overall analgesic duration, 24-h opioid consumption, patient satisfaction, or postpartum depression rates. These findings suggest that both blocks offer comparable postoperative analgesia, with ESP providing a marginal advantage in the immediate postoperative period. However, the clinical significance of this early difference warrants cautious interpretation, as it did not translate into broader benefits such as reduced opioid use or improved psychological outcomes.

Cesarean section can cause moderate to severe postoperative pain [12]. This pain consists of both somatic and visceral components. While TAP blocks are well established for somatic analgesia via abdominal wall nerve blockade (T10–L1), their limited visceral coverage has driven interest in alternatives like ESP blocks, which may anesthetize both somatic and visceral pathways [6,13]. Our results align with those of previous studies reporting comparable efficacy between ESP and TAP blocks in cesarean sections, such as that of Eksteen et al., where no differences in morphine consumption or satisfaction were observed [14]. However, our findings differ from those of Malawat et al., who reported superior pain control with ESP blocks at multiple timepoints, potentially due to weight-based dosing of local anesthetics [15]. In our study, a statistically significant difference in VAS scores was observed only at the 1st postoperative hour. Despite the absence of significant differences at later time points, the ESP group required fewer rescue analgesics and consumed 40 mg less tramadol over 24 h compared to the TAP group. This early and sustained analgesic benefit may be attributed to the dual somatic and visceral coverage provided by the ESP block, whereas the TAP block predominantly provides somatic analgesia. Beyond the early postoperative period, ESP and TAP blocks exhibited similar overall analgesic efficacy, underscoring their comparable roles in pain management after cesarean section. The prolonged analgesia duration of 15 h in our study compared to prior reported durations, such as 12 h in the study conducted by Boules et al. [16], may reflect the targeting of the T11 vertebral level, which aligns more closely with surgical dermatomes. This anatomical precision could enhance local anesthetic spread, though further cadaveric studies are needed to validate this hypothesis.

In the literature, the T9 level is generally preferred for ESP blocks in postoperative cesarean analgesia [15,17–20]. Aygun et al. applied a bilateral lower thoracic ESP block at the T11 level using a method similar to ours, and adding this to multimodal analgesia after cesarean section improved pain management compared to the control group and significantly reduced opioid requirements during the first 24 h [21]. We chose the T11 level for the ESP block, and it is also considered safer regarding the risk of pneumothorax. Although the ESP block is primarily considered a sensory block, unintended motor block was reported in a case at the T11 level [22]. However, in our study, no motor block was observed in any patient. This discrepancy may be due to differences in patient populations, local anesthetic concentrations, or individual anatomical variations. The increased sensitivity to local anesthetics during pregnancy might also influence the spread and effect of the block [23]. Therefore, local anesthetic dosing should be planned carefully for pregnant patients.

Yılmaz et al. compared postoperative analgesia in 90 patients undergoing elective cesarean section among ESP, TAP, and control groups. A total of 40 mL of local anesthetic solution (20 mL per side) comprising 0.25% bupivacaine and 0.5% lidocaine was administered bilaterally for both ESP and TAP blocks [24]. The inclusion of a control group in their study provided a clearer understanding of the effectiveness of block methods and allowed for a more objective evaluation of their impact on pain scores, analgesic use, and patient satisfaction. In contrast, the lack of a control group in our study limits the ability to make such definitive comparisons.

PPD, according to DSM-5 criteria, is defined as the onset of depressive symptoms during pregnancy or within the first 4 weeks postpartum. However, previous studies have indicated that the risk of developing PPD can extend beyond this period, particularly when persistent pain, hormonal fluctuations, and psychosocial stressors are present [25]. The relationship between postoperative pain and PPD has been highlighted in previous studies, suggesting that insufficient pain control may contribute to depressive symptoms. Shen et al. concluded that higher postoperative pain scores were significantly associated with an increased risk of PPD, suggesting that inadequate pain control beyond the acute postoperative period may contribute to PPD development [26]. In our study, we aimed to manage pain after cesarean section using ESP and TAP nerve blocks and assess their potential impact on PPD incidence. Postpartum depression as indicated by an EPDS score of ≥13 was observed in 13.04% of patients in the ESP group and 30.43% in the TAP group. Compared to other studies, our ESP group had a lower PPD rate; for example, the epidural group in the study by Tan et al., where an EPDS cutoff of ≥13 was also used, had a PPD rate of 15.9% [27]. Metaanalyses have demonstrated that ketamine and esketamine can reduce PPD by improving postoperative pain control [28]. Postpartum pain, particularly when involving both somatic and visceral components, can activate stress-induced inflammatory responses. Elevated proinflammatory cytokines such as IL-6 and TNF-α, along with hypothalamic–pituitary–adrenal axis dysregulation, have been implicated in increased susceptibility to PPD [29,30]. Improved postoperative pain control may help modulate these biological pathways and reduce the risk of PPD. Considering that the ESP block has been shown to provide both somatic and visceral analgesia, it is plausible that its more comprehensive pain relief contributed to the lower incidence of PPD observed in the ESP group, although this association remains speculative and requires further investigation.

This study has several limitations. First, the absence of a control group (e.g., with no block or with systemic analgesia alone) restricts the ability to determine the absolute efficacy of each technique. Second, the use of EPDS as a screening tool rather than a diagnostic instrument may have led to the overestimation or misclassification of depression rates. Third, the single-center design and homogeneous patient population limit generalizability to diverse settings. Fourth, the routine administration of 1 g of intravenous paracetamol to all patients may have contributed to overall analgesia and limited the ability to detect subtle differences in opioid requirements between groups.

Despite these limitations, our findings support the clinical equivalence of ESP and TAP blocks for analgesia after cesarean section. The choice between techniques may depend on clinician expertise, patient anatomy, or institutional protocols. For example, ESP blocks may be preferable in settings where early mobilization is prioritized, given their superior early pain control. Future research should explore optimized dosing regimens (e.g., weight-adjusted volumes), incorporate control groups, and employ longitudinal designs to evaluate long-term outcomes such as chronic pain and breastfeeding success. Additionally, integrating biomarkers or validated diagnostic tools for PPD could clarify the relationship between analgesia and mental health.

In conclusion, both ESP and TAP blocks are viable options for analgesia after cesarean section, with the ESP block offering a potential advantage in early pain control. While the observed benefits in rescue analgesic use may reflect enhanced visceral and somatic coverage with the ESP block, the overall similarity in patient satisfaction and depression outcomes underscores the clinical interchangeability of these techniques. Future studies with larger cohorts and extended follow-up are warranted to further clarify the role of regional anesthesia techniques in maternal recovery and mental health.

## Figures and Tables

**Table 1:** Sociodemographic and clinical data of the patients.

		Group E	Group T	p*

**Age (years), mean±SD**		29.10 ± 4.54	28.70 ± 5.20	0.752^a^

**Weight (kg), mean±SD**		75.50 ± 9.59	78.83 ± 12.36	0.248^a^

**Height (cm), mean±SD**		163.57 ± 4.35	162.00 ± 4.93	0.197^a^

**BMI (kg/m** **^2^** **), mean±SD**		28.24 ± 3.63	30.04 ± 4.64	0.101^a^

**ASA score, n (%)**	I	30 (%100)	30 (100%)	>0.9^b^
	II	0 (0%)	0 (0%)	

**Education Level**	Primary school, n (%)	7 (23.33)	7.00 (23.33)	0.757^b^

	High school, n (%)	10 (33.33)	12 (40)	

	University, n (%)	13 (43.33)	10 (33.33)	

	Graduate, n (%)	0 (0)	1 (3.33)	

**Comorbidities**	None, n (%)	25 (83.3)	20 (66.7)	0.86^c^

	GDM, n (%)	3 (10)	4 (13.3)	

	Hypothyroidism, n (%)	1 (3.3)	5 (16.7)	

	Arrhythmia, n (%)	0 (0)	1 (3.3)	

	Asthma, n (%)	1 (3.3)	0 (0)	

**Gestational period (days), median (Q1–Q3)**		271.5 (268.00–273.00)	271 (266.00–273.00)	0.744^d^

**Number of pregnancies, n (%)**	1	8 (26.7%)	8 (26.7%)	0.536^b^

	2	9 (30%)	12 (40%)	

	3	10 (33.3%)	5 (16.7%)	

	4	3 (10%)	3 (10%)	

	5	0 (0%)	1 (3.3%)	

	6	0 (0%)	1 (3.3%)	

**APGAR 1 min, median (Q1–Q3)**		8.00 (8.00–9.00)	9.00 (8.00–9.00)	0.145^d^

**APGAR 5 min, median (Q1–Q3)**		9.00 (9.00–10.00)	10.00 (9.00–10.00)	0.223^d^

a Independent-samples t-test;

b chi-square test;

c Fisher exact test;

d Mann–Whitney U test; α: 0.05;

* statistical significance between groups.

Group E: Erector spinae plane block group; Group T: transversus abdominis plane block group; SD: standard deviation; ASA: American Society of Anesthesiologists; APGAR: Appearance, Pulse, Grimace, Activity, and Respiration, a scoring system used to assess the health status of newborns at 1 and 5 min after birth; BMI: body mass index; GDM: gestational diabetes mellitus.

**Table 2:** Comparison of postoperative pain, analgesia, and patient satisfaction.

		Group E	Group T	p*
**Operation duration (min), median (Q1–Q3)**		52.5 (50–60)	60 (50–60)	0.276^a^
**Block analgesia duration (h), median (Q1**–**Q3)**		15 (14–15)	14 (14–15)	0.314^a^
**Total PCA consumption at 24 h (mg), mean±SD**		204.67 ± 102.75	241.33 ± 101.70	0.170^b^
**Number of rescue analgesics, median (Q1**–**Q3)**		0 (0–1)	1 (0–1)	**0.049** * a
**VAS pain scores, median (Q1–Q3)**	0th h VAS	0 (0–0)	0 (0–0)	1.00^a^
	1st h VAS	2 (2–2)	3 (2–4)	**0.032** * a
	6th h VAS	3 (3–4)	3 (3–4)	0.187^a^
	12th h VAS	3 (3–4)	3 (3–4)	0.591^a^
	18th h VAS	5 (4–6)	5 (4–6)	0.697^a^
	24th h VAS	6 (6–6)	6 (5–7)	0.874^a^
**CS scores, median (Q1–Q3)**	0th h CS	0 (0–0)	0 (0–0)	1.00^a^
	1st h CS	1 (1–1)	1 (1–1)	0.452^a^
	6th h CS	1 (1–1)	1 (1–2)	0.567^a^
	12th h CS	1 (1–1)	1 (1–2)	0.563^a^
	18th h CS	2 (2–2)	2 (2–2)	0.205^a^
	24th h CS	2 (2–3)	3 (2–3)	0.531^a^

a Mann–Whitney U test;

b independent-samples t-test; α: 0.05;

* statistical significance between groups.

VAS and CS scores were analyzed using the Friedman test (p < 0.001 for both), followed by Bonferroni post hoc comparisons to evaluate changes over time within groups. Group E: Erector spinae plane block group; Group T: transversus abdominis plane block group; SD: standard deviation; PCA: patient-controlled analgesia; VAS: visual analog scale; CS: comfort scale.

**Table 3:** Comparison of patient satisfaction and depression status.

		Group E	Group T	p*
**Patient satisfaction**	Very good, n (%)	22 (73.33)	17 (56.67)	0.336^a^
	Good, n (%)	7 (23.33)	10 (33.33)	
	Fair, n (%)	1 (3.33)	3 (10)	
**Complications**	Yes, n (%)	0 (0)	0 (0)	-b
	No, n (%)	30 (100)	30 (100)	
**Preop EPDS score**		7.93 ± 4.39	8.17 ± 3.83	0.827^c^
**Postop EPDS score**		9.39 ± 3.39	9.78 ± 6.44	0.798^c^
**Postpartum Depression (EPDS ≥13)**	Yes, n (%)	3 (13.04)	7 (30.43)	0.153^a^
	No, n (%)	20 (86.96)	16 (69.57)	

a Chi-square test;

b Fisher exact test;

c Mann–Whitney U test; α: 0.05;

* statistical significance between groups.

Group E: Erector spinae plane block group; Group T: transversus abdominis plane block group; EPDS: Edinburgh Postpartum Depression Scale; Preop: preoperative; Postop: postoperative.
